# Cars, compounds and containers: Judicial and extrajudicial infrastructures of punishment in the ‘old’ and ‘new’ South Africa

**DOI:** 10.1177/14624745221079456

**Published:** 2022-02-28

**Authors:** Gail Super

**Affiliations:** Department of Sociology, 71637 University of Toronto – Mississauga, Mississauga, Ontario, Canada

**Keywords:** Vigilante violence, extrajudicial punishment, forcible confinement, kidnapping, South Africa, colonialism, penal history, carceral geography, penal infrastructures, inequality, legal pluralism

## Abstract

This paper examines non-state infrastructures of vigilante violence in marginalized spaces in South Africa. I argue that car trunks, shacks, containers, and other everyday receptacles function as the underside of official institutions, such as prisons and police lock-ups, and bear historical imprints of the extrajudicial punishments inflicted on black bodies during colonialism and apartheid. I focus on two techniques: forcing someone into the trunk of a vehicle and driving them around to locate stolen property, and confinement in garages, shacks, containers, or local public spaces. Whereas in formerly ‘whites only’ areas, residents have access to insurance, guards, gated communities, fortified fences, and well-resourced neighbourhood watches, in former black townships and informal settlements, this is not the case. Here, the boot, the shack, the shed, the car, and the minibus taxi play multiple roles, including as vectors and spaces of confinement, torture, and execution. Thus, spatiotemporality affects both how penal forms permeate space and time, and how space and time constitute penal forms. These vigilante kidnappings and forcible confinements are not mere instances of gratuitous violence. Instead, they mimic, distort, and amplify the violence that underpins the state's unrealized monopoly over the violence inherent in its claims to police and punish.

## Introduction

This paper examines the role of everyday infrastructures of vigilante violence in former black townships and informal settlements in South Africa. I argue that car trunks, shacks, containers, and other commonplace receptacles function as the underside of official institutions, such as prisons and police lock-ups, and that they bear deep historical imprints and institutional legacies of the extrajudicial punishments that were inflicted on black bodies during colonialism and apartheid. They also serve as important reminders of the uneasy relationship between local meanings (and manifestations) of ‘justice’ and liberal legality.

Drawing on secondary historical literature, police records from two policing clusters in the Western Cape for the period 2000–2016, and selected court case transcripts, I situate my case study within the context of the penal violence exercised against racialized subjects during colonial and apartheid rule. I focus on two specific techniques: firstly, when someone is forced into the trunk of a vehicle and driven around in order to locate stolen property, and secondly, when a person is temporarily detained, assaulted and/or tortured in a garage, shack, container, or local public space. Since there is no statutory offence of vigilantism in South African law, this form of ‘self-help’ is framed in the police database as the common law offence of kidnapping (colloquially referred to as ‘manstealing’), and is usually combined with alternative charges of murder, attempted murder, and/or assault with intention to cause grievous bodily harm (GBH). Like police torture, it is more of a furtive and private ritual than a public act. As such it differs from spectacular incidents of collective violence, where a suspected offender is publicly burnt, stoned, or beaten to death by a crowd. While kidnapping as a technology of organized or politically motivated crime is a much studied phenomenon, it has been relatively neglected and undertheorized as a form of vigilantism, both in South Africa and beyond. It raises important questions about the overlaps between organized crime and crime as a ‘moralistic’ form of ‘social control’ (Black, 1983:34).

### Analytical framework and method

While Punishment and Society scholars tend to study particular institutions and spaces where punishment is imposed by the state, usually after a conviction in a criminal court, this paper questions the boundaries of the field. I use the term *extrajudicial* to refer to penal violence that is inflicted beyond the courts. From a strictly legalistic perspective this is not punishment (Zedner, 2016). However, in practice it is very much like punishment (Beckett and Herbert, 2010; Super, 2020). Certainly, those on the receiving end, and sometimes the instigators themselves, regard it as punishment. Like law, extrajudicial punishment assumes distinct forms and plays out differently, depending on where in space and time it is inflicted (Merry, 2004; Valverde, 2008). As such, it is multiscalar and spatiotemporally plural. This multiscalarity is highly visible in historically marginalized spaces and in contexts of inequality, South Africa being a case in point. In South Africa the law itself created spatial exclusions and spaces of marginality via legislation which not only established, but also compelled, most citizens to live in racially segregated black and ‘coloured’ townships. Thus, spatial forms of economic and social inequality are more marked in South Africa than elsewhere and historical methods are valuable tools for contextualizing this spatiotemporality.

Although kidnapping and the associated violence meted out against suspected criminals seems at first glance to be the opposite of lawfully exercised state power, I argue that it reflects, amplifies, and distorts the violence that is exercised by the state in support of its claimed monopoly over lawful (and hence, from the state's point of view legitimate) penal power. The extrajudicial civilian led penal violence that I discuss in the paper exists in close proximity to state practices of policing and punishment.^1^ Indeed, as Tazzioli and De Genova (2020:874) write

all forms of incarceration or detention involve some of the defining features of kidnapping, and it is strictly the often dubious distinction regarding what is a ‘lawful’ rather than an ‘unlawful’ purpose that separates the crime of kidnapping from these state practices of coercively taking a person into custody, spatially and temporally confining her, and depriving her of her liberty.

While official (i.e. state) punishment relies on the infrastructure of the criminal justice system (prisons, police vehicles, police cells, lock-ups, handcuffs etc.) those who inflict extrajudicial punishment in ‘subaltern spaces’ (Crush, 1994:314) have to tap into infrastructures which are located in or on the margins of the state. These infrastructures, and specifically the way they are used to inflict extrajudicial punishment, are the products (or spatial forms) of past and current social relations. As such they are invested with historical meaning and are spatialized.

The paper argues that non-state infrastructures such as the container, trunk, minibus, and car, are liminal or shadow spaces (generic containers) which are ‘temporarily converted’ (Yea, 2016:964) to achieve a punitive purpose by non-state actors in marginalized spaces. These infrastructures play key roles in what Jensen et al. (2017:7) refer to as the ‘violent exchanges’ which constitute the ‘everyday side of public authority’. The term ‘public authority’ (Lund, 2006) refers to non-state actors and/or non-state institutions who/which engage in state-like activities, such as meting out extrajudicial punishment against those accused of criminality or deviance. The vigilante kidnappings and extrajudicial spaces of confinement that I discuss, although formally illegal, mimic the practices, forms, and technologies of the state's penal and police power. At the same time they also amplify and distort state violence, bringing to mind the concept of ‘subversive mimesis’ (Feldman, 1991:178). Imitation (mimesis) is subversive (and can therefore function as a tactic of resistance) when it appropriates and subverts the original. Through ‘over-identification’ (Arns and Sasse, 2006:405) it reveals the obscenity (in this case the violence which underpins the state's lawful power to punish and police) while also producing more violence.^2^

While the carceral geography and crimmigration literature has generated productive discussions on how police lock-ups, cars, and trucks (such as the Black Maria discussed by Daly in this issue) are spaces of ‘pre-prison detention’ (Gear, 2021:57) or forms of ‘coercive mobilisation’ (Tazzioli and De Genova, 2020:875) this paper focuses on non-state infrastructures. I anchor my argument about how everyday objects are transformed into temporary infrastructures of extrajudicial punishment, which amplify and mimetically exaggerate the violence of state confinement and associated ‘disciplinary tactics’ (Tazzioli and De Genova, 2020: 874) in the socio-historic context of legal pluralism and penal violence which characterized colonial and apartheid rule (Brown, 2002; Chanock, 2001; Mamdani, 1996; Merry, 2004).

In making the argument that these are the underside or shadow of formal infrastructures of punishment I do not claim an equivalency. I am not arguing that there is a direct (or linear) connection between the prison and the trunk of a car, or a shack in an informal settlement, nor even that there is ‘fluidity between concepts of confinement’ (Armstrong and Jefferson, 2017:261), but rather that there is ‘fluidity between the quality of penal power itself’ (*ibid.)*, and the use of violence against those who threaten or violate ‘societal norms’.

The primary research for this paper consisted of an analysis of the computerized South African Police (SAPS) Crime Administration System (CAS) for the period 2000–2016, in respect of the Khayelitsha and Nyanga policing clusters in the Western Cape. Within these clusters I analyzed data from the Khayelitsha, Lingelethu-West, Harare, Nyanga, Gugulethu, and Philippi East police stations. These stations have among the highest recorded rates of violent crime in the Western Cape (if not the country) and are notorious for incidents of lethal vigilante violence (Department of Community Safety, 2019). By selecting data from these violent archives I risk sensationalising what are in essence tragic fragments of people's lives (Ross, 2005). However, there is also violence in not knowing, and in not exposing, what many in South African society (and beyond) turn a blind eye to. The SAPS shared their data with me via Excel spreadsheets, with each case having between 55 and 73 informational columns. I conducted an initial automated search to find cases that were potentially vigilante related. I then read through the ‘comments’ columns of the cases which the initial search had flagged, in order to determine whether these cases were connected to the punishment, prevention, or investigation of crime. I used specific search terms to conduct my initial automated search (see Appendix A for a list of the terms) and I focused on the generic crimes of Arson, Assault GBH, Attempted Murder, Malicious Damage to Property, Kidnapping, Public Violence, and Murder. Being a massive data set, dependent on human input, there was missing data across each offence category. Significantly, the police database only includes *recorded* cases, whereas, in practice, very few people are arrested for vigilante related violence, and many cases are not reported to the police, or if they are, the police fail to record them (O’Regan and Pikoli, 2014). Thus, I was able to get a sense of trends over time, but not exact figures.^3^ Since I did not follow up my analysis with specific interviews about vigilante kidnappings, the paper is written from an exploratory, rather than definitive, departure point. My intention is to highlight certain key themes that can be explored in future research.

## Repertoires of legal pluralism and ‘penal excess’^4^: Colonial legacies of contemporary practices

Patterns of racialized and spatialized violence, both formal and informal, have a long history in South Africa, with the legacies of colonial and apartheid-era violence shaping the structural inequalities and social relations that underpin contemporary penal practices. The ‘form of statehood’ that European colonizers imposed onto African colonies was different to that in the metropole (Bierschenk, 2014:224). The colonial state was mainly concerned with extracting natural resources and it relied on exploitation, violence, and political control (rather than an effective bureaucracy) as central pillars of rule (Nugent, 2010). Thus, penal excess against racially subordinate populations, rather than accountability, buttressed its rule (Brown, 2002; Bierschenk, 2014). The myth of ‘civilized’ whites versus ‘savage’ blacks underpinned the narrative and practices of corporal punishment as a means to control black subjects (Chanock, 2001; Pavlich, 2018). Whether official (meted out by the state) or unofficial (in the form of the random and arbitrary violence imposed by white settlers on black subjects), corporal punishment was a central instrument of racialized and paternalistic control (Alexander and Kynoch, 2011; Glaser, 2018). It was an integral mechanism for the ‘civilizing mission’ in terms of an ethos which framed Africans as ‘child-like’, only able to comprehend the language of bodily violence, and ruled by customary law because they were ‘not yet ready for autonomy’ (Alexander and Kynoch, 2011: 400; Chanock, 2001: 34).

Tactics of penal violence and criminalization combined with processes of expulsion and dispossession to produce spaces of marginalization. In this context, infrastructures such as compounds, mines, townships, and hostels were either carceral in design (to facilitate surveillance and control) and/or they permitted a plethora of actors to punish lawfully, in support of, or justified by, the pursuit of summary justice. These included mining companies, *indunas* (‘commanders’), *baasboys* (‘bossboys’), chiefs, native commissioners, and white farmers. Summary [in]justice was further enabled by poorly trained administrative officials who exercised judicial, legislative, and executive powers against black subjects, mostly via regulation and hence beyond the reach of judicial oversight (Chanock, 2001; Bierschenk, 2014:224). As Chanock (2001:2) points out, this pluralist legal approach explicitly sanctioned violence and patriarchal power relations, and subjected black Africans to an ‘extensive, localized and legally arbitrary [form of] rule’. The courts generally accepted the evidence of white experts, who embraced an authoritarian view of customary law, presenting all chiefs as having the same absolute powers and customs, able to ‘act without consultation or consent’ (Chanock, 2001: 288). Thus, local chiefs and headmen could impose unreviewable orders and fines, along with imprisonment for non-compliance, over their subjects (Chanock, 2001: 289). Traditional heads of rural households could also lawfully inflict ‘reasonable’ corporal punishment for the purposes of discipline.

Closed compounds, mining hostels, and black townships were designed to maximise surveillance and control, keep black and white labour separate, and ensure a steady supply of cheap labour (Nieftagodien, 2017; Crush, 1994; Mamdani, 1996). As Crush (1994:307) argues, the carceral architecture of the first mining compounds provided ‘important clues about the spatial exercise of power’ in this ‘fusion of prison and mining infrastructures’ (Gill et al., 2018:194). Within these spaces (and elsewhere) there was a thrust towards summary ‘justice’ for ‘natives’, with penal violence being an essential part of this ‘justice’. Apart from the power of summary arrest over anyone who defied his (they were invariably male) authority, native commissioners were given delegated powers of punishment which were considered ‘administrative acts’ and hence not subject to judicial review (Chanock, 2001: 289). Mining compounds had *stokkies* (small wooden huts) where miners could be ‘locked up, isolated and punished for a few days for minor offences’ (Crush, 1994:310), and it was standard for black workers to be detained without pay after their contracts ended - to ensure they had not stolen any diamonds. White farmers also used to lock up their labourers at night. These forms of detention were illegal, but largely accepted (Chanock, 2001: 435). The use of violence to control wage labourers by *baasboys* on farms, and *indunas* on mines, was common.

Like mining compounds, black townships were designed to enable a ‘maximum degree of control with the least amount of effort’ (Mills, 1989:72). These racially segregated spaces were to be situated as far as possible from white neighbourhoods but close to the industrial areas so that they could be containers of cheap labour (Smit, 2016). There were a minimum number of access roads to the white city. As such the black township was almost like an enclave (or a prison)^5^ – ‘a point of origin or destination’, but not lying along a ‘through route’ (Mills, 1989:67). The ‘geometrically formal’ internally identical street pattern, which was ‘intelligible’ from above, resulted in a series of ‘isolated domains’ in which it was easy for visitors to get lost (Mills, 1989:72). This ‘regimented spatial system’ was conducive to the commission of violent crimes because of the spatially structured lack of ‘surveillance by householders or moving people’ (ibid.). It also provided the means ‘whereby large numbers of people could be concentrated together and easily controlled’ (ibid).

This racialized political and legal fragmentation deepened once the National Party gained power in 1948. It poured enormous resources into continuing the forcible relocation of black people to the ‘urban periphery’ (Smit, 2016: 37) and to *bantustans* (‘homelands’) in impoverished rural areas. This resulted in violent ‘local social orders’ (Bénit-Gbaffou, 2008: 96) with splintered jurisdictions where violence played, and continues to play, a central role in the exercise of both state and non-state sovereignty (Kynoch, 2011; Glaser, 2000, 2018). The apartheid state was more concerned with protecting white citizens from the supposed criminal threat presented by black people, than it was with the prevention of crime inside black townships and in *bantustans* (Super, 2010). Hence, there were very few police stations in black townships. Township residents were forced to rely on inter-personal and patronage networks as forms of security, and vigilante associations were common.

By the time the first democratic elections were held in 1994, there was a deep historical legacy of instant justice in which violence played a central role (see e.g. Buur, 2005; Glaser, 2000; Hund and Kotu-Rammopo, 1983; Super, 2017). Corporal punishment was not only meted out by state officials and settlers but was also a central technology of discipline and identity formation among the subjects of apartheid rule. As a ‘gendered instrument of generation[al] control’ (Glaser, 2018:2), it was particularly aimed at keeping young men in their place (Crais, 1998). During the 1980s when township activists (many of them youth), supported by the African National Congress in exile, embarked on a campaign of ungovernability, longstanding patriarchal networks of authority started to fracture (Marks, 2008; Gibbs, 2014) with new forms and networks of vigilantism and popular justice emerging. Not only was the line between ‘political’ and ‘ordinary’ crime a blurred and shifting one (Super, 2010, 2016) but popular justice initiatives sometimes collapsed into violent punishments for ordinary criminal offences, such as theft and the use of drugs, as opposed to being targeted at collaborators and apartheid spies (*impimpis*). Beatings were inflicted by some people's courts as a mode of social control for those deemed counter-revolutionary (Buur, 2005; Crais, 1998; Alexander and Kynoch, 2011; Marks and McKenzie, 2001; Super, 2017). Liberation movements in exile also relied heavily on flogging as a disciplinary technique and harsh punishments were meted out against deviants in the camps (Alexander and Kynoch, 2011; Kynoch, 2011). Offenders were sometimes tied to trees or locked in windowless containers as part of their punishment (Ngculu, 2009, Skweyiya, 1992, in Super, 2013:115).

These legacies of colonial and apartheid-era violence, what Thomas (2011:124) refers to as historic ‘techniques of embodied violence’, still exist as ‘potential resources’ in South Africa. As such they provide a ‘template’ or ‘repertoire’ for violence in the current era (Thomas, 2011: 124), even though the targets have changed. As Feldman (1991), writing about Northern Ireland argues, when the state uses violence and dirty tactics against those Others, which it has discursively brought into existence by, for example, creating the legal category of ‘terrorist’ and/or criminalizing political resistance, it ends up producing an ‘Other’. As he writes (1991:178): the production of ‘the Other (of the state) is always the detached part of the state that has been invested with alterity’. The state then reclaims itself by deploying expulsive violence against this Other, resulting in violence amplification and distortion. This scenario played out in South Africa during the 1980s, when the National Party government resorted to increasingly violent and underhand tactics to fight against ‘*die swaart*’ and ‘*rooi gevaar*’ (the ‘black’ and ‘red’ danger). There were about 2000 enforced disappearances under apartheid, most of them perpetrated by members of the Police Security Branch and Counter-Insurgency Unit (Sarkin, 2015). The case of Simelane, a young anti-apartheid activist who was last seen in the trunk of a policeman's car in 1983, tortured and detained at a block of flats, and thereafter at a farm in the northern part of the country, is one example of how cars, boots, flats and farms were deployed as infrastructures in the disappearances (and extrajudicial punishments) secretly carried out by the State in the name of ‘security’.^6^ Vlakplaas Farm, where shadowy state agents perpetrated multiple executions, torture, and unlawful confinements of anti-apartheid activists, is another. This infliction of horrific and deeply punitive violence by apartheid state operatives produced ‘violent subjectivities’ (Rueedi, 2015:403) inside township communities. This shaped counter-violence against *impimpis*, the police, the hated community councillors, and other apartheid collaborators (Rueedi, 2015; Super, 2010). Petrol bombing, arson, stoning, and necklacing were far more common than abductions and torture, although some counter-violence did include these technologies. In this way then, to use Feldman's (1991:178) words, the state's ‘construction of the Other’ resulted in a very real ‘transfer of force, an empowering investiture of alterity’ that was ‘played back against the state’ in such a way that the ‘copy’ affected the ‘model’. This essentially means that the state's excessive violence creates forms of resistance that distort, mimic, and exaggerate the state's original violence. This engenders further spirals of violence or, ‘proliferating violent reciprocity’, hence precipitating ‘new social forms’ of violence which become ‘autonomous, culturally generative, and meaning endowed’ practices (Feldman, 1991:158).

Next, I discuss how everyday infrastructures such as vehicles, shacks, and containers, function as the underside or shadow of the state's infrastructures of violence, now deployed against the criminal (as opposed to the political) Other in the service of extrajudicial punishment. This violence, which emerges ‘in the peripheries of the infrastructures of state power’(Roitman, 2004:144), while appearing to be gratuitous, confirms the ‘right and logic of extant modes of thinking and enacting power’ (*ibid.*) and is subversively mimetic. It not only confirms the logic of state punishment, rendering its violence explicit, but also challenges the state's claim to monopolize it.

## Spaces of mobility, ‘stuckness’,^7^ and death

The post-1994 South African state has embraced community policing alongside punitive and exclusionary discourses and practices of crime control. This has resulted in ‘racial banishment’ (Roy, 2018) in wealthy and gentrifying areas, and punitive forms of local ‘justice’ in former black townships (Bénit-Gbaffou, 2008: 106; Super, 2013, 2016). In both rich and poor areas it is the unemployed young black man who is accused of criminality, and hence vulnerable to violence by both the police and others (Jensen et al., 2017).^8^ Affluent people living in formerly ‘whites only’ areas enjoy the benefits of resources and infrastructures such as private security; electrified fences; gated communities; insurance policies; vehicles; police stations^9^; and social capital which facilitates access to the criminal justice system. In marginalized spaces, however, where residents are economically and politically trapped in their immediate spaces and disconnected from the ‘networks of spatial links’ (Pillay, 2008: 150) that are available to the affluent, everyday infrastructures and objects are adapted to serve multiple purposes, including that of punishment. Thus, a piece of wire can be transformed into a handcuff, a boot into the back of a police vehicle, a beach into a death or torture chamber. The possibilities are endless.

The police database reflects this unequal spatiality, suggesting that ‘black lives and deaths’ (Gillespie, 2015: 204) are largely invisible in the greater context of the city. To give two illustrative examples: in one case someone was accused of stealing his neighbour's property and forcibly taken to a community hall, which was temporarily transformed into a ‘people's court’. After female participants were ordered to leave the room it morphed into a violent interrogation centre in which the ‘accused’ was suffocated with a plastic bag, strangled, and beaten with a plastic *sjambok* (whip).^10^ Despite the severity of the assault the police comments record ‘no serious injuries’ (KA). In another case (KB) a young man was abducted and assaulted so badly that he had to spend a week in hospital with an intercostal drain in his chest He was beaten with a firearm, wood, and hammer, forced into the ‘boot’ of a small car and driven around to find stolen property. He took his abductors to a ‘container’ which doubled up as a shop for selling stolen goods but, since it was closed, his assailants forced his head under a tap and continued to beat him. Thereafter they wrapped him in a blanket, put him back in the trunk, and drove him to another container where they intended to incarcerate him for the night. The plan was thwarted when the police arrived on the scene and heard him screaming. Two years later, after a process of court ordered mediation, the charges against his assailants were withdrawn on the condition that they apologised and paid him 1500 ZAR (about 100 US$). A hundred dollars for a week in hospital, a broken leg, stitches in his head, back, face, and a collapsed lung. It is worth pausing to consider whether this would have happened had the person not been a young black male from a marginalized former black township?

Resources such as firearms and smart cars, used by organized crime (or gangsters) also play central roles in the service of supposedly ‘combatting’ crime, tracing stolen goods and/or punishing those accused of criminality. In a substantial number of cases both the ‘vigilantes’ and the ‘criminals’ had criminal records. In one case six men, armed with firearms, kicked down the door of a shack at 3 a.m. and accused the two male occupants of having stolen a plasma television. After being driven around and heavily assaulted (burnt with an iron, having boiling water thrown over them), one man died before the assailants realized that they were ‘assaulting the wrong people’. The assaulters (now categorized as ‘suspects’ in the police notes) had withdrawn theft, fraud, and assault charges on their records, and one had a conviction for unlawful possession of a firearm (GA). In another case, a person convicted of vigilante violence had a string of previous convictions: for Malicious Damage to Property, two convictions of housebreaking, assault, a liquor license contravention, and possession of stolen property (KC). Such cases demonstrate that the boundaries between vigilantes who purportedly act against crime and other organized (and also less organized) networks or formations of violence, are blurred and porous.

### Discipline, punish, and drive (sometimes to the beach)

Cars, which are ordinarily conceptualised as vectors of mobility, also serve as spaces of confinement and instruments of punishment. Until the 1960s and 1970s, when car ownership grew amongst the better-paid black workers, public transport consisted of an inadequate state-owned bus and rail infrastructure. In the 1970s informal sedan taxis (it was almost impossible for a black person to get a taxi license) began to fill the transport gap, with illegal minibuses emerging in the 1980s (Gibbs, 2014; Bank, 1990; Sekhonyane and Dugard, 2004). From the start the minibus taxi industry was fragmented and mired in violence. Taxi owners had to secure protection against police harassment and against their competitors. This produced networks of violent exchange between taxi owners, the state, street gangs, and vigilantes (Bank, 1990). In the mid-1980s, due partly to the consumer and bus boycotts, and attacks on trains and buses, the state started to relinquish its monopoly over public transport. By the late 1980s minibus taxis rivalled buses and trains ‘as the primary form of commuter and long-distance transport’ (Gibbs, 2014: 435).

Given an overall lack of insurance cover in former black townships and informal settlements, recovering stolen property is central to residents’ sense of justice, and having access to a vehicle renders this more feasible. However, since car ownership is still relatively low in these marginalized ‘high risk areas’ (to use insurance company terminology), the owners or drivers of minibus taxis (colloquially known as ‘Quantums’^11^ or ‘tatas’) play a key role in providing these ‘services’ (Buur, 2005; Gibbs, 2014, Jensen et al., 2017; Super, 2016), which ‘come at a price’ (Jensen et al., 2017:14). As ‘exemplars of patriarchal authority’ (Gibbs, 2014: 433–434), with a reputation for violence, taxi-owners have the means to ‘bring together and summon an entourage’ (Gibbs, 2014: 444). Vehicle ownership and mobility are obviously central to this successful image.

In 2012 the Western Cape Minister of Community Safety praised Khayelitsha's ‘taxi bosses’ for assisting the police in solving and preventing crimes (Mackay, 2012) and a high ranking police official acknowledged that taxi associations were ‘in control of vigilante actions’, resulting in a ‘decrease in crime’ which made ‘everyone (including the police) …very happy’(De Kock, 2012: 4). In the context of a police force which lacks sufficient vehicles with which to investigate crimes, it is unsurprising that the ubiquitous Quantum minibuses, whose drivers and owners are in an excellent position to engage in a variety of ‘transactions, trades, and relationships’ (Gibbs, 2014: 443), sometimes function as quasi-police vehicles, tracking down stolen goods and collecting evidence. This explains why the term ‘Quantum’ came up repeatedly in the police database, although I also found less organized instances of sedan style vehicles (a red Golf, a black BMW, a white Toyota Corolla, a blue Polo etc.) which were temporarily transformed into vectors of violence.

In these marginalized spaces, vehicles function as crucial infrastructures of mobility, spaces of confinement, discipline, and spectres of death - particularly when someone is taken away and never seen again. The sequence of events plays out along these lines: a car with approximately six men^12^ stops in front of a home, they ask about the whereabouts of stolen property, or of a person, and leave with someone in the trunk. Being cramped inside the windowless space of a ‘boot’ has disciplinary, retributive and instrumental effects. While the vehicle itself is a vector for travelling from point A to B, the effect of being confined inside the trunk not only disciplines the captive into confessing but also raises the very real possibility of death. As such it functions as a ‘space of transformation’ (Buur, 2005:201), a space where a human being is changed into an Other, into someone against whom ‘a formidable right to punish is established’ (Foucault, 1995:90). Thus, when a Quantum arrives unsummoned outside someone's house, in the dead of night, it is indeed like a spectre, something ominous and foreboding. Seeing a vehicle full of people is a harbinger of the transformation (through violence) to come.

In one case, ten men drove off with a 23 year old, who lived in a shack in his father's backyard, at 10 p.m. He was never seen again (NA). Sometimes victims are tied to the towbar and dragged behind a car. One complainant was woken up at 02h00 a.m. by a group of taxi drivers searching for his brother who had allegedly broken into people's houses. They dragged him to a ‘white Toyota Quantum’ where he was beaten with a firearm and wheel spanner on his head and body, causing him to lose consciousness. They then bound his legs together with a rope, tied the one end to the towbar, and drove off (HA).

Sometimes the extractive nature of the violence is blatantly obvious, as in the case where five men kicked down the door to a shack, forced two brothers into their Quantum, drove them to the taxi rank and punched, *sjambokked* and stabbed them with a screw driver, because the brothers had allegedly stolen their tires. They ended up paying their abductors 4000 ZAR (275$), even though they denied stealing the tires (PEA). Indeed, as Buur (2005) argues, vigilantes do not care whether the property that they ‘find’ is the stolen property or not – as long as they get something. In another case after a stolen DVD player and amplifier were found in a shack, the vigilantes proceeded to take ‘everything they wanted’ (Z) - they took far more than the value of the stolen goods.

Sometimes people are driven to Monwabisi Beach, a public space reserved for ‘blacks only’ during apartheid. It borders the western side of Khayelitsha and is on a stunning but dangerous stretch of the Atlantic coastline. When people are forcibly taken there at night, it is temporarily transformed from a space of leisure into a space of violence and death. In one case, a 23 year old was abducted while he was at work. He was driven to a graveyard and thereafter to the beach where he was tortured for a prolonged period, in retaliation for having robbed a cellphone and wristwatch from someone in an informal settlement a few days previously. During the five hour assault, he was struck with bricks, stabbed with sharp objects, burnt with molten plastic, forced to drink sea water and eat sand, taken back to the graveyard, and left for dead (GB). In another case two brothers (accused of stealing their neighbour's laptop) were brutally assaulted with *sjamboks*, forced into a car and driven around to locate the computer (even though they denied the accusations). Their abductors threatened to throw them into the sea, which due to strong currents and because many residents in former black townships cannot swim, is akin to a death sentence.

### Containers of punishment

While the common garden shed might serve as a space to keep tools and gardening equipment in the leafy middle class suburbs, in poor former black townships and informal settlements those who cannot access formal bricks and mortar houses live in corrugated iron shacks (*ihoki*) and in garden sheds (colloquially known as ‘Wendy houses’, or in Xhosa, *ityotyombe*). They also double up as spaces of incarceration – for the purpose of punishment and torture. In one instance a police officer found two people inside a locked shed in someone's backyard. One of them was semi-conscious, unable to walk or speak, with multiple wounds (GC). The other had been beaten and burnt with molten plastic all over his body because the owner of the house suspected they were thieves and had, presumably, been torturing them in order to get information. One photograph in a police docket showed a garage which had been temporarily converted into a torture chamber. The police had made markings on the photograph around the bloodstains on a wooden chair in the centre, and at hooks on the wall (PR). Here mechanical instruments, such as chains and winches, doubled up as instruments of torture and confinement.

These processes both mirror and distort the central role that arrest and interrogation play in the construction of state power, and they ‘mobilize the state as spectacle’ (Feldman, 1991:88) when they mimic its’ search and seizure practices. In former black townships and informal settlements, the accusation or ‘the moment where accusers …allege that there are criminal outsiders’ occurs in various spaces, *none of which* are ‘expressly designed to hear accusations’ (Pavlich, 2018: 14). Sometimes, however, the state's spaces and infrastructures of accusation coalesce with the spatiotemporal multipurpose penal infrastructures discussed here. For example, where a charge of shoplifting was withdrawn after the ‘accused’ had been arrested and appeared in court, the victim of the shoplifting offered him a lift home but instead drove to another house. Here, he was interrogated about the whereabouts of the stolen items, burnt with a hot iron and beaten by four men, before being forced into the trunk of the vehicle and driven to ‘point out the belongings.’ They found nothing (NB). In another case, where someone was accused of stealing 800 ZAR (55$) from his cousin's wallet, the accuser called her boyfriend who, upon arrival, assaulted the ‘suspect’, drove him somewhere else where he continued the assault, and thereafter dropped him off at the police station. Despite being a victim of vigilantism, he (i.e. the theft suspect) was handcuffed and arrested by the police (KE). The police do not always co-operate however, as I discovered when I interviewed a member of an informal community crime prevention group in 2014. John (not his real name) related how he had, together with others, arrested 24 people one night and locked them in some toilets, before calling the police, who only arrived some four hours later - at 3 a.m. As he bitterly remarked: ‘The police accuse us and tell us it's illegal. They brought them back to us after five minutes, in the same amount of time it took me to open a coke … the (police) van never stopped and told us why they were releasing them’.

It is viscerally obvious, when focusing on the material infrastructures which non-state actors draw on to exercise penal power, that extrajudicial policing (including torture) and punishment are prone to collapse into each other. Yet, in South Africa (and elsewhere) communities are encouraged to take responsibility for their own policing, and citizens’ arrests are lawful (Bénit-Gbaffou, 2008; Super, 2016). Thus, for example, after ‘community patrollers’ arrested two people who were attempting to break into a house, they detained them in a ‘green container’. They then drove them to a taxi rank, where they handcuffed one victim's hands together and beat him with a *sjambok* on his naked body (S). In another case, where someone had initially gone to the police to report that his cellphone had been stolen he thereafter, at the investigating officer's recommendation, approached an ‘anti-crime committee’ for assistance in retrieving the phone. The committee members forced a suspect into the boot of a vehicle and took him to a container that served as their offices. Because he ‘did not want to tell the truth’^13^ the suspect was held in the container for what a witness described as ‘a long time’ and was violently interrogated by members of the committee. He eventually led his interrogators to someone who had a phone that was the same colour as the stolen one, and identified it as the theft victim's phone.

Thus, while forcible confinement in a shack (or other container) does not have the authority of the law, and is not a prison or police lock-up in the legal sense of the term, I have argued that it fulfills a similar, albeit exaggerated function to that of lawful incarceration. In a context where few have access to justice ‘unofficial confinements’ are an ‘immediate and tangible form of punishment’ - substituting for ‘complicated, slow, and costly judicial processes’ (Kyed, 2020:8).^14^

## Conclusion

Colonial and apartheid rule encouraged a plethora of actors to punish extrajudicially. Whether through the technologies of torture, corporal punishment, and/or judicial and extra-judicial killings, black lives were deemed infinitely expendable and state-sanctioned forms of violent ‘non-state’ punishments were central techniques of rule. This resulted in deeply-rooted and multiscalar violent extrajudicial punishments which have shaped current repertoires of violence and violent forms of extrajudicial punishment. These are not the opposite of, but instead constitute the underside, and always present, twin of liberal penality. Thus, contrary to liberal discourse on state punishment, (extrajudicial) punishment is not a post-conviction event but an ongoing process, in which torture and unofficial confinement are central elements. It starts well before conviction (if there is a conviction at all).

As Benjamin (1996) notes, law is founded on, and preserved through, violence. South Africa, because of its history of racialized dispossession and segregation, which played key roles in producing and/or or providing a repertoire of, and template for, current forms of penal violence, presents an excellent case study of this relationship between law and its violent ‘other’. The expulsive spatial practices, which were legislated for in law, resulted in a deeply spatialized form of legal pluralism. This spatialization plays out in the ways that everyday infrastructures are used to fulfil multiple functions, including penal ones. Whereas in middle class, formerly white only areas, residents have access to police stations, insurance policies, security guards, gated communities, fortified fences, and well-resourced neighbourhood watches, in former black townships and informal settlements, where there is endemic poverty, this is manifestly not the case. Here instead it is the boot, the shack, the shed, the car, and the minibus taxi which play multiple roles, including as vectors and spaces of confinement, torture, and execution. Thus, spatiotemporality is crucial to both how penal forms permeate space and time, and to how space and time constitute penal forms. As such the field of Punishment and Society should broaden the concept of punishment, to incorporate what occurs in practice, as opposed to restricting it to what should happen, in terms of the narrow confines of liberal theory. The latter completely fails to acknowledge, in fact it obscures, the multiscalarity of liberal penal forms. The vigilante kidnappings and forcible confinements discussed in this paper are not mere instances of gratuitous violence. Instead, they mimic, distort, and amplify the violence that underpins the state's unrealized monopoly over the violence inherent in its own claims to police and punish.

At the same time, however, it is not always possible to tease apart the reasons and motivations behind lawful and unlawful punitive violence. The same (or similar) technologies and infrastructures are deployed for multiple, sometimes conflicting, purposes. Just as the boundaries between state and non-state punishment are porous, so too are those between vigilantism and organized (or less organized) crime. This becomes particularly obvious when vigilantes themselves have criminal records. This finding has implications for the mainstream, ahistorical, and reductionist claim that ‘inefficient’ criminal justice systems produce vigilantism (Sekhonyane and Louw, 2002; Swanepoel et al., 2011). The term ‘vigilantism’ itself is an umbrella for a host of violent forms, many of which are deeply rooted in historic practices of state violence. Thus, future research should investigate the nuances and distinctions, the similarities and overlaps, between vigilantism, ordinary crime, organized crime, and lawful state violence.

Racialized forms of structural inequality, high rates of violent crime, widespread poverty, and a neoliberal call by the state to devolve responsibility for crime prevention to ‘communities’ at a local level play key roles in the production of extrajudicial punishment in South Africa. These punitive forms of local justice, and their infrastructures, are the effect of the segregation that produced these inequalities (Hutta, 2019: 71). The forms of violence enabled and produced by the colonial and apartheid states did not just disappear, particularly since the new South African state embraced a punitive approach to crime, which was unevenly implemented (Bénit-Gbaffou, 2008). This unevenness was both a product of, and a contributor towards, spatial inequality. The strong tradition of racialized alterity in South Africa is an outcome of the expulsions and dispossessions that were central to colonialism and apartheid and the continuing inequality which they produced. Although the generic ‘criminal’ has now replaced the ‘terrorist’ and the *impimpi*, penal violence targeted at poor racialized people continues. As such, it is important to study the infrastructures of extrajudicial punishment precisely because these are not merely objects but ‘lively sites of meaning making and struggle in their own right’ (Walters, 2015: 483).

## Notes

1. Contrary to liberal legal discourse the boundaries between policing and punishment are, in practice, porous. The police often engage in extrajudicial punishment and civilian policing often collapses into extrajudicial punishment.

2. Arns and Sasse (2006: 405) use the term ‘subversive affirmation’ to describe participation for the purposes of undermining. This results in a ‘revelation of what is being affirmed’ because ‘there is always a [destabilising] surplus which turns affirmation ‘into its opposite’ (ibid.).

3. See Super, 2021 for detail on methodology and numbers.

4. This term comes from Brown (2002: 404).

5. See Wacquant (2001) on the parallels between prisons and ghettos. See also Jefferson et al. (2018).

6. During this period the South African Police separated out ‘the combatting of crime’ from the maintenance of law and order’ (Super, 2013: 90), concentrating on violently quelling political resistance in black townships, while devolving crime prevention to the ineffective and despised black municipal authorities and municipal police.

7. This term comes from Jefferson et al, 2018.

8. Although gender based violence is endemic in South Africa the police database contained only two cases where women were the targets of vigilante kidnappings (one was combined with a rape).

9. There are (still) proportionately fewer police stations in former black townships than in formerly white areas (O’Regan and Pikoli, 2014).

10. Street committees do not openly impose or sanction violence. Hence, interrogation is conducted outside by a few men, or after the women are ordered outside (Super, 2016).

11. The ‘Quantum’ is manufactured by Toyota. Used colloquially it means an illegally refurbished panel van, or the more expensive specially designed (to carry between 10–12 people) passenger vehicle.

12. Few cases record female participation in kidnappings.

13. *S v Hena & another* 2006 2 SACR 33 (SE).

14. The boundaries between torturing ‘just enough’ to gain information and violent extrajudicial punishment are so porous as to render the distinction between them inane. There are of course numerous examples of the police and other state agents engaging in exactly the same practices.

## References

[bibr1-14624745221079456] Alexander J Kynoch G (2011) Introduction: histories and legacies of punishment in Southern Africa. Journal of Southern African Studies 37(3): 395–413.

[bibr2-14624745221079456] Armstrong S Jefferson A (2017) Disavowing ‘the’ prison. In: Moran D Schliehe AK (eds) Confined Places, Secure Spaces: The Spatialisation of Studies of Confinement. Palgrave Studies in Prisons and Penology. Basingstoke: Palgrave Macmillan, pp. 237–269.

[bibr3-14624745221079456] Arns I Sasse S (2006) Subversive affirmation: On mimesis as a strategy of resistance. East Art Map, Contemporary Art and Eastern Europe. London, Cambridge: MIT Press, 444–456.

[bibr4-14624745221079456] Bank L (1990) The making of the Qwa Qwa “mafia”? Patronage and protection in the migrant taxi business. African Studies 49(1): 71–93.

[bibr5-14624745221079456] Beckett K Herbert S (2010) Banished: The New Social Control in Urban America. New York: Oxford University Press.

[bibr6-14624745221079456] Bénit-Gbaffou C (2008) Community policing and disputed norms for local social control in post-apartheid johannesburg. Journal of Southern African Studies 34(1): 93–109.

[bibr7-14624745221079456] Benjamin W (1996) Critique of violence. In: Bullock M Jennings M (eds) Selected Writings, vol. 1, 1913–1926. Cambridge, MA: Harvard University Press, 236–252.

[bibr8-14624745221079456] Bierschenk T (2014) Sedimentation, fragmentation and normative double-binds in (West) African public services. In: Bierschenk T De Sardan O (eds) States at Work. Dynamics of African Bureaucracies. Leiden, Boston: Brill Press, 221–249.

[bibr9-14624745221079456] Black D (1983) Crime as social control. American Sociological Review 48(1): 34–45.

[bibr10-14624745221079456] Brown M (2002) The politics of penal excess and the echo of colonial penality. Punishment and Society 4(4): 404–423.

[bibr11-14624745221079456] Buur L (2005) The sovereign outsourced: local justice and violence in Port Elizabeth. In: Blom Hansen T Stepputat F (eds) Sovereign Bodies. Citizens, Migrants, and States in the Postcolonial World. Durham: Duke University Press, pp. 192–217.

[bibr12-14624745221079456] Chanock M (2001) The Making of South African Legal Culture 1902–1936: Fear, Favour and Prejudice. Cambridge: Cambridge University Press.

[bibr13-14624745221079456] Crais C (1998) Of men, magic, and the law: popular justice and the political imagination in South Africa. Journal of Social History 32: 49–72.

[bibr14-14624745221079456] Crush J (1994) Scripting the compound: power and space in the South African mining industry. Environment and Planning: Society and Space 12: 301–324.

[bibr55-14624745221079456] De Kock C (2012) Serious crime in khayelitsha and surrounding areas, crime research, crime intelligence. *Affidavit of Major-General De Kock (AL30)* 3 August 2012. Available at: http:///www.khayelitshacommission.org.za/images/highcourtpapers/282 (accessed 15 April 2014).

[bibr15-14624745221079456] Department of Community Safety (2019) *Western Cape Crime Report for 2018-2019*. https://www.westerncape.gov.za/sites/www.westerncape.gov.za/files/wccs_crime_report_2020-03-25 (accessed 2 April 2020).

[bibr16-14624745221079456] Feldman A (1991) Formations of Violence, Body and Political Terror in Northern Ireland. Chicago and London: University of Chicago Press.

[bibr17-14624745221079456] Foucault M (1995) Discipline and Punish, The Birth of the Prison (Transl. Alan Sheridan, 1977). New York: Vintage Books.

[bibr18-14624745221079456] Gear S (2021) ‘As if they can squeeze you to death’, recollections of post-arrest journeys towards and into prison in South Africa. In: Morelle M Le Marcis F Hornberger J (eds) Confinement, Punishment and Prisons in Africa. Routledge.

[bibr19-14624745221079456] Gibbs T (2014) Becoming a “big man” in Neo-liberal South Africa: migrant masculinities in the minibus-taxi industry’. African Affairs 113(452): 431–448.

[bibr20-14624745221079456] Gill N Conlon D Moran D, et al. (2018) Carceral circuitry: New directions in carceral geography. Progress in Human Geography 42(2): 183–204.

[bibr21-14624745221079456] Gillespie K (2015) Murder and the whole city. Anthropology Southern Africa 37(3–4): 203–212.

[bibr22-14624745221079456] Glaser C (2000) Bo-tsotsi: The Youth Gangs of Soweto, 1935–1976. Cape Town: James Currey.

[bibr23-14624745221079456] Glaser C (2018) Nostalgia for a beating: discipline, generational authority and corporal punishment at a Soweto high school, c. 1960–2000. History of Education 48(3): 395–409.

[bibr24-14624745221079456] Hund J Kotu-Rammopo M (1983) Justice in a South African township: The sociology of Makgotla. Comparative and International Law Journal of Southern Africa 16(2): 179–208.

[bibr25-14624745221079456] Hutta J (2019) From sovereignty to technologies of dependency: rethinking the power relations supporting violence in Brazil. Political Geography 69: 65–76.

[bibr26-14624745221079456] Jefferson A Turner S Jensen S (2018) Introduction: On stuckness and sites of confinement. Ethnos 84(1): 1–13.

[bibr27-14624745221079456] Jensen S Andersen M Larsen K, et al. (2017) Introduction: towards violent exchange. In: Jensen S Anderson M (eds) Corruption and Torture: Violent Exchange and Policing of the Urban Poor. Aalborg: Aalborg University Press, pp. 5–39.

[bibr28-14624745221079456] Kyed HM (2020) Provisional police authority in Maputo’s inner-city periphery. Environment and Planning D: Society and Space 38(3): 528–545.

[bibr29-14624745221079456] Kynoch G (2011) ‘Of compounds and cellblocks: The foundations of violence in Johannesburg, 1890s −1950s. Journal of Southern African Studies 37(3): 463–477.

[bibr30-14624745221079456] Lund C (2006) Twilight institutions: public authority and local politics in Africa. Development and Change 37(4): 685–705.

[bibr56-14624745221079456] Mackay M (2012) Plato talks tough against vigilante attacks. Sowetan, 3 May. Ref:4004, SA Media.

[bibr31-14624745221079456] Mamdani M (1996) Citizen and Subject: Contemporary Africa and the Legacy of Late Colonialism. Cape Town: David Philip.

[bibr32-14624745221079456] Marks M (2008) Young Warriors. Youth Politics, Identity and Violence in South Africa. Johannesburg: Wits University Press.

[bibr33-14624745221079456] Marks M McKenzie P (2001) Alternative policing structures? A look at youth defence structures in Gauteng. In: Schärf W Nina D (eds) The Other Law: Non-State Ordering in South Africa. Lansdowne: Juta.

[bibr34-14624745221079456] Merry S (2004) ‘Colonial and postcolonial law’. In: Sarat A (ed) The Blackwell Companion to Law and Society. Malden, Oxford, Victoria: Blackwell, 569–589.

[bibr35-14624745221079456] Mills G (1989) Space and power in South Africa: The township as a mechanism of control. Ekistics; Reviews. on the Problems and Science of Human Settlements 56(334/335): 65–74.

[bibr57-14624745221079456] Ngculu J (2009) The Honour to Serve: Recollections of an Umkhonto Soldier. Cape Town: David Philip.

[bibr36-14624745221079456] Nieftagodien N (2017) ‘Life in South Africa’s hostels: carceral spaces and places of refuge’. Comparative Studies of South Asia, Africa and the Middle East 37(3): 427–436.

[bibr37-14624745221079456] Nugent P (2010) States and social contracts in Africa. New Left Review 63: 35–68.

[bibr38-14624745221079456] O’Regan K and Pikoli V (2014) Towards a safer Khayelitsha. Report of the Commission of Inquiry into Allegations of Police Inefficiency and a Breakdown in Relations between SAPS and the Community of Khayelitsha, August. Available at: https://www.westerncape.gov.za/policeombudsman/files/atoms/fileskhayelitsha_commission_report_0.pdf. (accessed on 3 February 2022).

[bibr58-14624745221079456] Pavlich G (2018) Criminal Accusation: Political Rationales and Socio-Legal Practices. London: Routledge.

[bibr39-14624745221079456] Pillay S (2008) Crime, community and the governance of violence in post-apartheid South Africa. Politikon 35(2): 141–158.

[bibr40-14624745221079456] Roitman J (2004) Power is not sovereign: The pluralization of economic regulatory authority in the Chad Basin. In: Hibou B (ed) Privatising the State. London: Hirst, 120–146.

[bibr41-14624745221079456] Ross F (2005) Codes and dignity. Thinking about ethics in relation to research on violence. Anthropology Southern Africa 28(3&4): 99–107.

[bibr42-14624745221079456] Roy A (2018) ‘At the Limits of Urban Theory: Racial Banishment in the Contemporary City’.

[bibr59-14624745221079456] Rueedi F (2015) Siyayinyova: patterns of violence in the African townships of the vaal triangle south Africa, 1980-1986. *Africa* 85(3): 395–416.

[bibr43-14624745221079456] Sarkin J (2015) ‘Dealing with enforced disappearances in South Africa (with a focus on the Nokuthula Simelane case) and around the World: The need to ensure progress on the rights to truth, justice and reparations in practice**’** (Vol 1) SPECJU 5 (http://www.saflii.org/za/journals/SPECJU/2015/5.html).

[bibr44-14624745221079456] Sekhonyane M Dugard J (2004) ‘A violent legacy. The taxi industry and government at loggerheads. South African Crime Quarterly 10: 13–18

[bibr45-14624745221079456] Sekhonyane M Louw A (2002) ‘Monograph 72: Violent Justice, Vigilantism and the State’s Response’. http://www.issafrica.org.

[bibr60-14624745221079456] Skweyiya L (1992) *Report of the Commission of Enquiry into Complaints by Former African National Congress Prisoners and Detainees*. Belleville: Published on behalf of the Commission by the Centre for Development Studies, University of the Western Cape.

[bibr46-14624745221079456] Smit W (2016) ‘Informal settlement upgrading: international lessons and local challenges’. In: Cirolia L Görgens T van Donk M Smit W Drimie S (eds) Upgrading Informal Settlements in South Africa. A Partnership Based Approach. Cape Town: UCT Press, pp. 26–48.

[bibr61-14624745221079456] Super G (2010) The spectacle of crime in the “new” South Africa: a historical perspective (1976-2004). British Journal of Criminology 50(2): 165–184.

[bibr62-14624745221079456] Super G (2013) Governing Through Crime in South Africa: The Politics of Punishment and Race in Neo-Liberalizing Regimes. Burlington: Ashgate, Advances in Criminology series.

[bibr63-14624745221079456] Super G (2016) Volatile sovereignty: governing crime through the community in Khayelitsha. Law and Society Review 50(2): 450–483.

[bibr64-14624745221079456] Super G (2017) What’s in a name and why it matters: A historical analysis of the relationship between state authority, vigilantism, and penal power in South Africa. Theoretical Criminology 21(4): 512–531.

[bibr65-14624745221079456] Super G (2020) Three warnings and you’re out: banishment and precarious penality in South Africa’s informal settlements. Punishment and Society 22(1): 48–69.

[bibr66-14624745221079456] Super G (assisted by Avila, F. and Teran, J.) (2021) Report to SAPS: Vigilante Violence in the Khayelitsha and Nyanga Policing Clusters, 2000–2016. Unpublished on hand with author.

[bibr47-14624745221079456] Swanepoel M Duvenhage A Coetzee T (2011) ‘Vigilantism: A Theoretical Perspective as Applied to People’s Courts in Post-1994 South Africa’. http://www/dspace.nwu.za.

[bibr48-14624745221079456] Tazzioli M De Genova N (2020) Kidnapping migrants as a tactic of border enforcement. Society and Space 38(5): 867–886.

[bibr49-14624745221079456] Thomas D (2011) Exceptional Violence. Embodied Citizenship in Transnational Jamaica. Durham: Duke University Press.

[bibr50-14624745221079456] Valverde M (2008) Analyzing the governance of security: jurisdiction and scale. Behemoth: A Journal on Civilisation 1: 3–15.

[bibr51-14624745221079456] Wacquant L (2001) Deadly symbiosis: when ghetto and prison meet and mesh. Punishment & Society 3(1): 95–133.

[bibr52-14624745221079456] Walters W (2015) Migration, vehicles, and politics: three theses on viapolitics. European Journal of Social Theory 18(4): 469–488.

[bibr53-14624745221079456] Yea S (2016) Everyday spaces of human trafficking: (In)visibility and agency among trafficked women in U.S. Military-oriented clubs in South Korea. Annals of the American Association of Geographers 106(4): 957–973.

[bibr54-14624745221079456] Zedner L (2016) Penal subversions: when is a punishment not punishment, who decides on what grounds? Theoretical Criminology 20(1): 3–20.

